# Covid-19 in Cartagena and the Bolívar Department, Colombia. Current status, perspectives and challenges until the arrival of the vaccine

**DOI:** 10.1016/j.heliyon.2021.e06336

**Published:** 2021-02-19

**Authors:** Fabián Espitia-Almeida, Ramiro Pereira-Lentino, Juan Quintero-Soto, Doris Gómez-Camargo

**Affiliations:** a UNIMOL Research Group, School of Medicine, University of Cartagena. Cartagena de Indias 130001, Colombia; b Doctorate in Tropical Medicine, School of Medicine, University of Cartagena. Cartagena de Indias 130001, Colombia; c Departmental Laboratory of Public Health of Bolivar, Cartagena de Indias 130001, Colombia

**Keywords:** COVID-19, SARS-CoV-2, Vaccine, Surveillance, Pandemic

## Abstract

COVID-19, caused by SARS-CoV-2, a new coronavirus, was first observed in Wuhan (China) in November 2019. In a short time, SARS-CoV-2 spread across the world, creating a pandemic. There is a need to know the current situation of each country and region and to generate strategies to contain and mitigate the impact on global health and the economy. To control COVID-19 in Cartagena and the Department of Bolívar, Colombia, a strategic network involving public health entities and higher education institutions has emerged. The network has been in place for six months, and 77,122 subjects have been tested in Cartagena and Bolívar Department, of whom 8,260 (10.71%) tested positive (RT-qPCR). Of those who tested positive, 51.4% were male (*p>0.05*), and 13.1% were health personnel (9.43% female, *p < 0.05*). The mortality rate was relatively low, 1.22%, with males being the most affected, accounting for 0.9% of deaths (*p > 0.05*). The daily case report showed upward and downward fluctuations by the mobility restrictions applied to the population, and from day 120 of the start of the pandemic, the epidemiological curve stabilized, and a logarithmic plateau was reached. COVID-19 spread in 39/46 municipalities of Bolívar; however, Bolívar and Cartagena had a low number of cases and deaths compared to other departments and city in Colombia. Cartagena and Bolívar have been given an economic opening with restrictions on crowding and mandatory use of a mouth cover until a vaccine is available.

UNIMOL was the first laboratory in Cartagena, Bolívar and Colombia to receive approval from the National Institute of Health to process COVID-19 samples; thanks to the timely diagnosis of cases by UNIMOL, intensive care unit (ICU) occupancy did not exceed capacity, and population confinement was appropriately initiated.

## 1 Introduction

COVID-19 infection caused by the new severe acute respiratory syndrome coronavirus 2 (SARS-CoV-2) arises in Wuhan Province of Hubei (China) in November 2019, and at the end of the same year, it was reported as an infectious outbreak associated with the consumption of wild animals [1, 2, 3, 4]. The spread of the virus was so rapid that in a short time the disease spread worldwide according to reports from the World Health Organization (WHO), becoming a major threat to the physical and mental health of the population, leaving devastating results of mortality and reduction of the economy [3, 5, 6, 7, 8, 9], so it was made official as a pandemic on March 11, 2020 [5]. At the time of writing the manuscript (January 2020), 191 countries were affected, reporting >91 million infections and approximately 1,948,236 deaths. The highest burden of the disease is located on the American, Asian, and Europe continents [10], with the United States being the most affected (>22.6 million cases and 376,295 deaths), followed by India (>10.4 million cases and 151,327 deaths), Brazil (>8.1 million cases and 203,580 deaths), Russia (>3.4 million cases and 61,908 deaths), United Kingdom (>3.1 million cases and 82,096 deaths), France (>2.8 million cases and 68,198 deaths), Turkey (>2.3 million and 22,981 deaths), Italy (>2.2 million cases and 79,203 deaths), Spain (>2.1 million cases and 52,275 deaths, Germany (>1.9 million cases and 41,917 deaths) and Colombia (>1.8 million cases and 46,451 deaths). China, the epicenter of the pandemic, ranks 82th among the countries most affected by the virus, with 97,001 cases and 4,793 deaths [10].

In Colombia, COVID-19 entered on March 6, 2020 [11]. Since it was a new disease, health personnel, scientists and government entities were not prepared to fight it. At the beginning of the pandemic, the samples taken in the 32 departments of the country were transferred to the National Institute of Health (Instituto Nacional de Salud - INS) in Bogotá to be processed. The high volume of samples and the difficulty of moving them to Bogotá due to mobility restrictions made the delivery of results to be delayed and sometimes the samples were damaged during transport [12]. The need to process the samples in each department arose, generating strategies between academia, research and government entities, which gave rise to the Surveillance Network against COVID-19 in the Department of Bolívar. This network was formed by different entities of the Health sector, the Departmental Laboratory of Public Health of Bolívar, experts in collection, field trips, surveillance and control and the Molecular Research Unit (Unidad de Investigación Molecular - UNIMOL) Laboratory of the Universidad de Cartagena, experts in Molecular Biology and research tools. This alliance allowed reducing the time for delivery of results, establishing epidemiological fences and pandemic control in Cartagena and the Department of Bolívar.

## 2 Materials and methods

In a descriptive study of active epidemiological surveillance against COVID-19 in Cartagena and the Department of Bolivar, the subjects selected for the study were recruited through a multistage cluster sampling of unequal sizes between April 1 and November 30, 2020.

### 2.1 Selection criteria

To be part of the study, participants had to meet at least one of the following selection criteria: attend a health service with suspicious symptoms of COVID-19 (fever ≥38.5 °C, difficulty breathing, severe dry cough, loss of smell and taste, gastrointestinal symptoms), report previous contact with positive cases for COVID-19, be staff of the health sector, attend the sampling sessions conducted in Cartagena and the remainder municipalities of the department by the EPS and IPS.

### 2.2 Ethical aspects

The study was reviewed and approved by the Ethics Committee of the University of Cartagena; written informed consent was obtained; for minors, the consent of the responsible accompanying person was requested. This research is classified with minimal risk, according to the second classification of article 11 of ethical aspects of research in humans (resolution 8430 of 1993). The personal data of each subject were coded and archived in databases under the custody of the UNIMOL Laboratory and the Departmental Laboratory of Public Health of Bolivar.

### 2.3 Study population

Bolívar is a Department of the Colombian North Coast and has a population of approximately 1.9 million inhabitants distributed in 46 municipalities. Of these, approximately 1 million people are accumulated in the Cartagena Capital, which is the tourist, commercial and industrial center of the Department. The department is surrounded by extensive beaches and rivers, so its main economy is tourism, fishing, loading and unloading of ships (national and international) in seaports that receive and ship merchandise worldwide, which are optimal conditions for the entry and spread of the virus [13, 14].

### 2.4 Sampling and data collection

**Figure 1:**
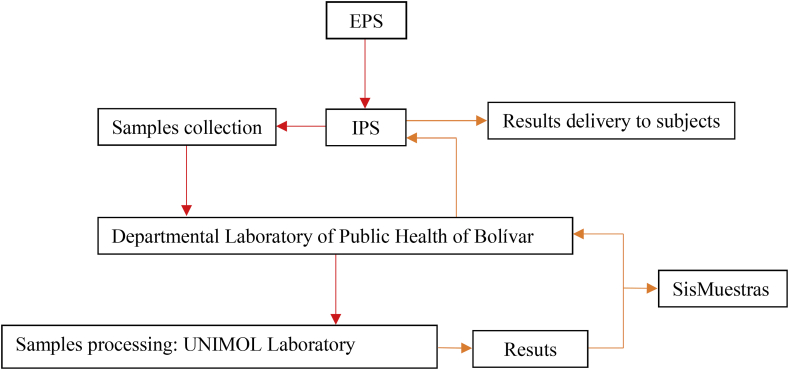
Surveillance network: process for sampling, transportation, processing and delivery of results.

A surveillance network was created between different actors of the Health, Education and Research sector in Cartagena and the Department of Bolívar, whose objective was the active search for positive cases of COVID-19 (Figure 1). The flow indicates the direction for the organization and takes of nasopharyngeal swab samples (red line) by the Health Provider Entities (Entidades Prestadoras de Salud - EPS) and the Service Provider Institutions (Instituciones Prestadores de Servicios - IPS), who sent the samples to the Departmental Public Health Laboratory and this in turn sent them to the UNIMOL Laboratory where they were processed free of charge. After the processing, the UNIMOL Laboratory uploaded the results to the platform for national registry of patients and samples (SisMuestras) within 24 h (orange line). The Departmental Laboratory of Public Health sent them to the IPS-EPS, and finally, these delivered the results to the patients. All this is controlled and supervised by the INS of Colombia, which validates the processes and results. The UNIMOL Laboratory complies with the parameters of quality and reproducibility of required results, obtaining 93/100 points in quality and reproducibility of results.

### 2.5 Extraction of viral RNA and detection of SARS-CoV-2 by RT-qPCR

The extraction of viral RNA was performed using the following kits: COVID-19 Viral RNA Extraction MagicMag Magnetic Beads (BioBasic), QIAamp Viral RNA mini Kit (Qiagen), PureLinnk Pro 96 Viral RNA/DNA Purification Kit (Invitrogen), Quick-RNA Viral Kit (Zymo Research), MGIEasy Nucleic Acid Extraction Kit (MGI) and COVID-19 Viral RNA Extraction ez-10 Spin Column (BioBasic). For all RNA extraction kits, the manufacturer's recommendations were followed. Detection of SARS-CoV-2 and diagnosis of COVID-19 were performed using the following kits: Genesig® Real-Time PCR assay (Primerdesign Ltd), COVID-19 RT-qPCR Detection (Bio-Speedy) and GeneFinder COVID-19 Plus RealAmp Kit (GeneFinder); adjusting the run protocol in the RT-qPCR to the instructions of each manufacturer.

### 2.6 Definition of cases

All subjects who tested positive for SARS-CoV-2 in the RT-qPCR test were called cases; the run-in considerations in the RT-qPCR for case confirmation were established following the indications of each kit, in general a threshold of 100 thousand copies and that the amplification occurred before cycle 37 (CT ≤ 37). Positive and negative controls and RNA controls were always processed.

### 2.7 Statistical analysis

The information obtained in the file of each subject was stored in a database developed in Excel and subsequently analyzed with SPSS software (version 25). Categorical variables were analyzed with absolute frequencies and relative frequencies. The numerical variables were first evaluated using the Kolmogorov–Smirnov statistic to determine their normality. When the numerical variable followed the assumption of normality, it was represented by the mean and standard deviation; when the numerical variable did not follow the normal distribution curve, it was analyzed by the median and interquartile range. To determine the significant differences between groups, a cutoff point of *p < 0.05* was used and evaluated using Fisher's two-tailed exact test and the chi-square test.

## 3 Results

The data and results described in this manuscript refer only and exclusively to the samples processed by the UNIMOL Laboratory from Cartagena and the Department of Bolívar between April 1 and November 30, 2020 (See supplementary material). It is important to note that at the beginning of the pandemic, the only laboratory that was processing COVID-19 samples in Cartagena and Bolivar was UNIMOL, and only until August were other laboratories of the department able to process COVID-19 samples. However, UNIMOL continues to be the laboratory that processes the most samples and works together with the Departmental Laboratory of Public Health of Bolivar for the control of the pandemic.

### 3.1 General description of the selected subjects

**Figure 2:**
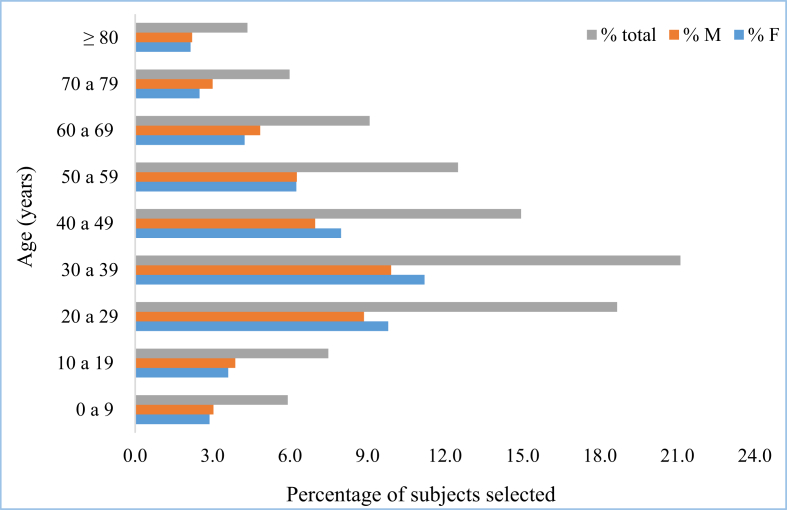
Subjects selected by age group and gender.

A total of 77,122 subjects were included, of which 37,671 (48.8%) were male and 39,451 (51.2%) were female, *p > 0.05*. The age represented as the median and interquartile range was 38 [55-26] years. A total of 54.7% (n = 42,186) of the selected subjects were between 20-49 years of age (Figure 2), and 46.5% (n = 35,862) reported an epidemiological history of risk from previous contact with active cases of COVID-19; of these, 10,605 (13.75%) were personnel from the health sector.

### 3.2 Description of cases

**Figure 3:**
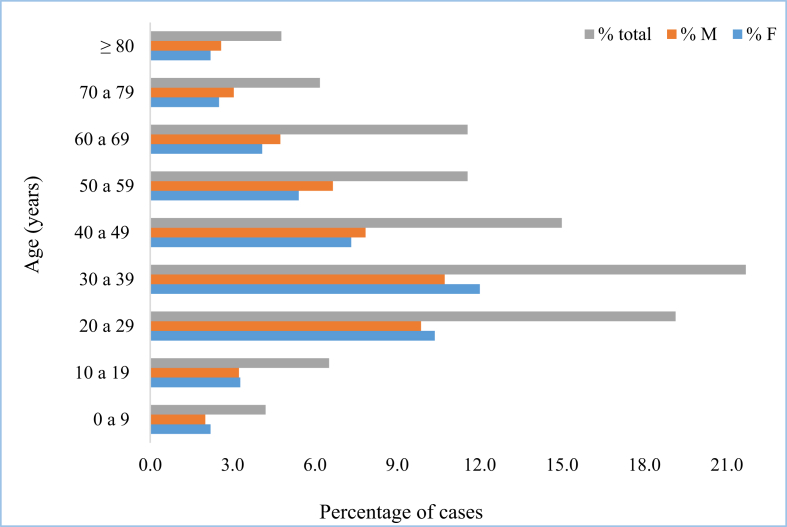
Cases by age and gender.

Of the total of subjects selected, 8,260 (10.71%) tested positive for COVID-19, distributed by gender in 4,244 (51.4%) males and 4,016 (48.6%) females, of which >90% were asymptomatic and recovered at home with direct support from the Ministry of Health of Bolivar. The median age and interquartile range were 38 [57-27] years, and the distribution by gender and age shows that cases of COVID-19 occurred more frequently in the population aged 20–49 years (Figure 3). The frequency of infection in the group aged 0–39 years was higher in the female gender; in contrast, the male gender had a higher frequency of infection in subjects aged ≥40 years.

In the cases, the diagnoses of medical consultation and on which the sampling was performed were: 88.3% respiratory disease by new coronavirus, 6.1% influenza-like disease, 4.5% acute respiratory infection and 1.1% others.

**Figure 4:**
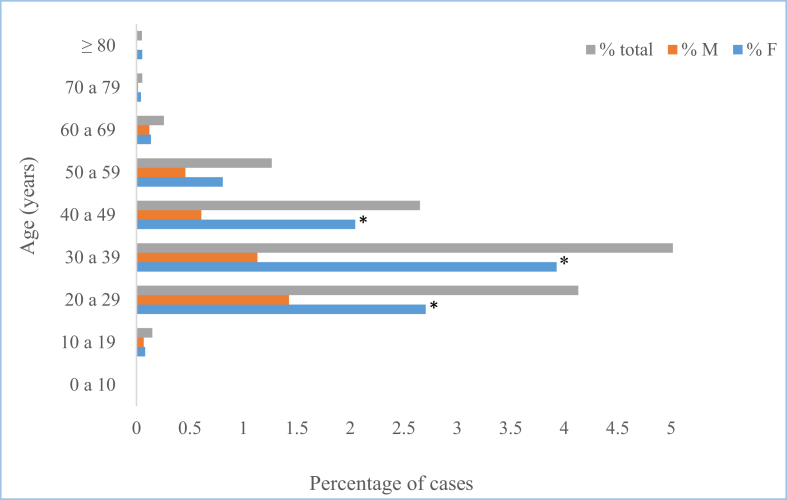
Health sector cases by gender and age. (∗) The differences were statistically significant as long as *p < 0.05*.

Personnel in the health sector were affected in a high proportion, with 1,082 (13.1%) of the positive cases, and the frequency of infection was significantly higher in females (9.43%) than in males (3.67%). *p < 0.05*. Additionally, the most affected age groups in healthcare personnel were 30–39, 20–29 and 40–49 years, with 12.04% of cases; for these three groups, the differences between the frequencies of infection in females and males were statistically significant (*p < 0.05*) (Figure 4).

#### 3.2.1 Distribution of cases by municipality

**Figure 5:**
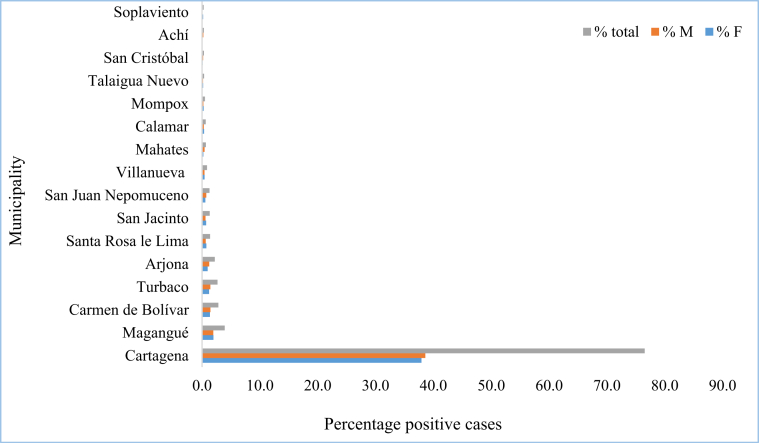
Cases by municipality and gender.

The virus spread in 36 of the 46 municipalities of the Department of Bolívar; the 16 most affected municipalities had frequencies between 0.31-76.52%, and the rest had frequencies <0.27%. The Cartagena capital of the department was the most affected, with 5,688 (76.52%) cases, followed by Carmen de Bolívar, Magangué, Turbaco, Arjona and Santa Rosa de Lima, with frequencies of 3.9, 2.8, 2.6, 2.2 and 1.4%, respectively. The male gender was affected to a greater degree in more than 95% of the municipalities (Figure 5).

#### 3.2.2 Report of cases per day and flattening of the epidemiological curve

**Figure 6:**
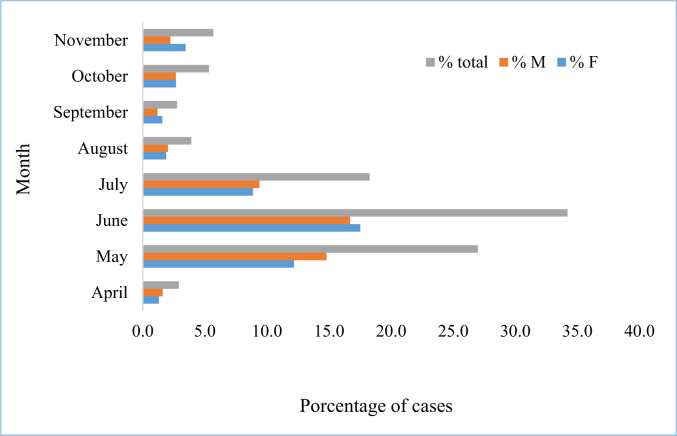
Cases by month and gender.

The months with the highest report of cases were June 2805 (39.8%), May 2202 (31.3) and July 1483 (21.0%), and the male gender presented the highest percentage of cases in all months, except in June, where greater involvement was observed in the female gender (Figure 6).

**Figure 7:**
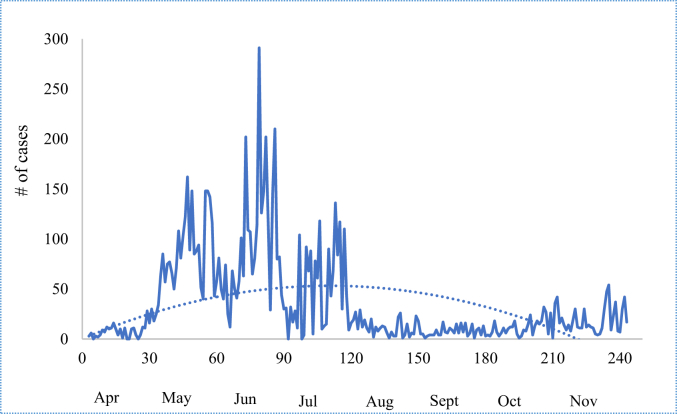
Daily case report.

**Figure 8:**
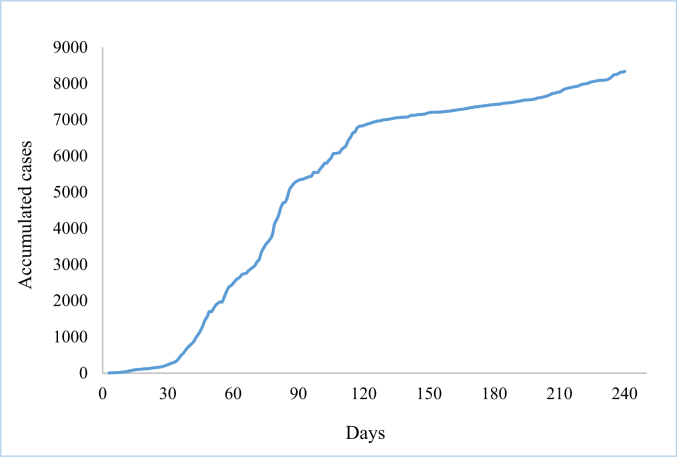
Epidemiological curve.

**Figure 9:**
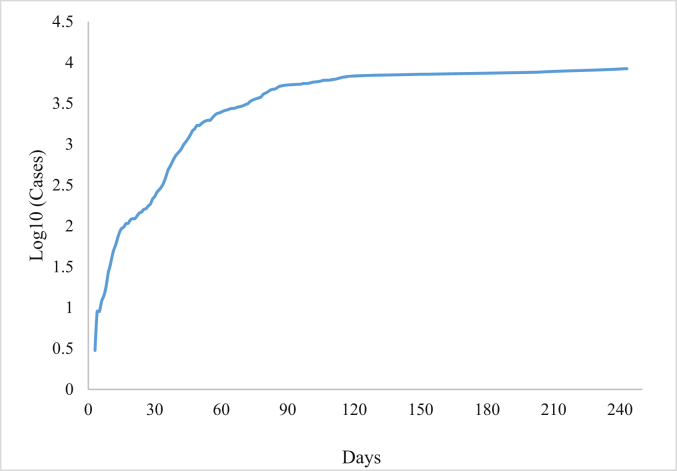
Logarithmic curve of the epidemiological behavior of the pandemic in the Department of Bolívar.

The report of new cases per day showed upward and downward fluctuations, mainly linked to the number of samples received for processing and the advance of the pandemic. The maximum peak of the pandemic in Cartagena and the Department of Bolívar was found in June, and from this point on, a substantial decrease in the reporting of daily cases was observed (Figure 7). Flattening of the epidemiological curve is observed from day 120 of having started the surveillance network (Figure 8). In addition, from days 30–120, the increase in accumulated cases increased exponentially and accelerated until 90th day (end of June and beginning of July) when the slowdown of the pandemic is evident, currently remains on a very stable plateau (Figure 9).

#### 3.2.3 Mortality of cases

**Figure 10:**
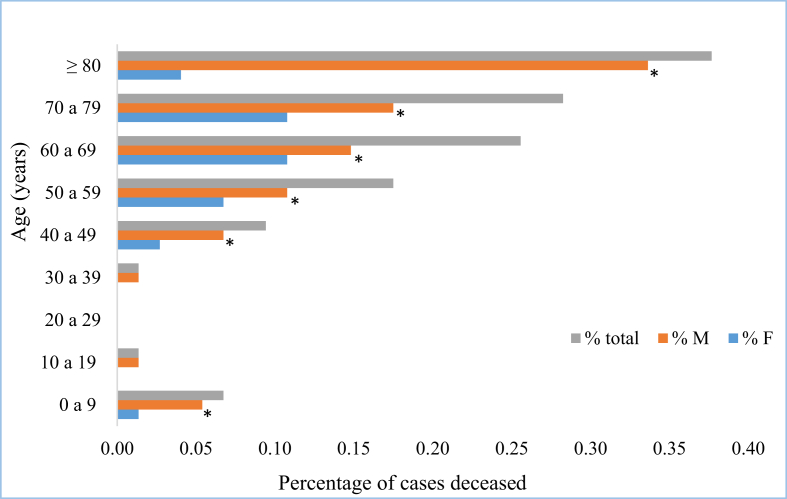
Mortality of cases by age and gender. (∗) *p < 0.05*.

The mortality of subjects caused directly the virus was 1.22% (n = 101), with a higher frequency of mortality in males (0.87%, n = 72) than in females (0.35%, n = 30) (*p < 0.05*). The age groups with the highest mortality were ≥80, 70–79 and 60–69 years, with 0.38, 0.28 and 0.26%, respectively (Figure 10). The frequency of mortality in children 0–9 years was 0.07%, and all were male. The group of subjects aged 20–29 years, despite being the second with more positive cases, did not present deaths, presenting itself as the group with the best prognosis for recovery. Mortality in health personnel was 0.024% (n = 2).

**Figure 11:**
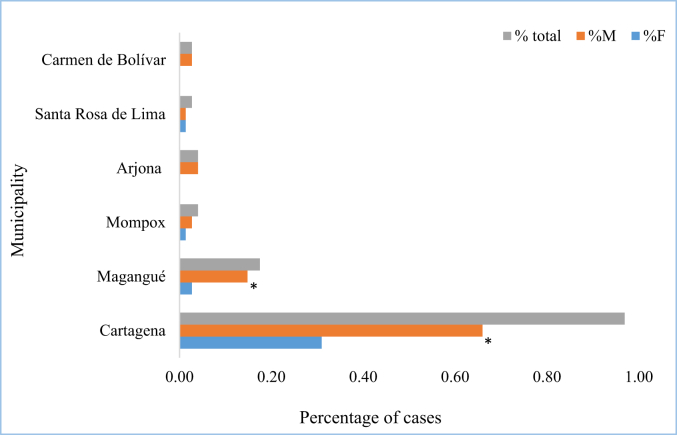
Deaths by municipality and gender. (∗) *p < 0.05*.

The deceased cases were distributed in 6 municipalities, with Cartagena, Magangué and Mompox being the most affected, with 0.97, 0.17 and 0.04% of the total deaths, respectively. El Carmen de Bolívar and Arjona did not present deaths in female subjects (Figure 11).

**Figure 12:**
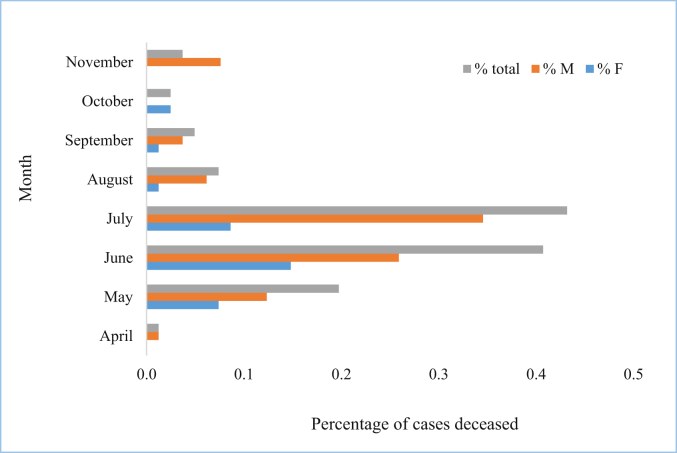
Mortality of cases per month. (∗) *p < 0.05*.

The months with the highest frequency of mortality were June 0.47% (n = 33) and July 0.45% (n = 35). In contrast, April and September had the lowest frequencies (0.01 and 0.05%, respectively) (Figure 12).

## 4 Discussion

This paper shows the current status of the COVID-19 pandemic in Cartagena and the Department of Bolivar, Colombia, over a period of six months (April 1 - November 30) and the prospects and future challenges. We did the active search of positive cases of COVID-19 in Cartagena y Bolívar, thanks to the strategic of prevention and control network against the COVID-19 formed between different actors of the health, research and government entities sector (Figure 1). The formation of this strategic network for the containment of the pandemic COVID-19 goes hand in hand with OECD global policies in Latin America that seek to reduce the impact generated by COVID-19 on the economy and human health [15]. The notification of the positive or negative result was done in less than 24 h, in all this process, when the delivery of results to patients lasted more than 24 h, it was because there was a delay in the transfer of samples to the Departmental Laboratory of Public Health and the UNIMOL Laboratory, or the EPS did not make timely notification of results to the patients.

In total, 77,123 subjects of different ages were included (Figure 2), of which 8,260 (10.71%) were positive. The cases in Bolívar compared to other departments of Colombia and the national behavior were relatively low (Table 1). The cumulative incidences per 100 thousand inhabitants were also compared to approximate the impact of cases in relation to the population size of each department, placing Bolívar in position 10 and well below Bogotá, Amazonas and Antioquia, which occupy the first places [16]. If this prevention and control strategy had not been applied, the current panorama in Bolivar would be similar to other departments with high infection rates.

**Table 1:** Cases and cumulative incidence in Bolívar and other Departments of Colombia [16].

Department	Population	Confirmed cases	Incidence of cases/100 thousand inhabitants
Bogota	7,181,000	374,077	5,209.3
Antioquia	6,855,517	212,758	3,103.4
Valle del Cauca	3,789,874	107,558	2,838.1
Santander	2,185,000	52,896	2,420.9
Cundinamarca	2,792,877	52,110	1,865.8
Atlantic	2,342,265	32,812	1,400.9
Goal	919,129	28,120	3,059.4
Nariño	1,335,521	25,414	1,902.9
**Bolivar**^a^	**1,909,460**	**8,260**	**432.6**
Amazon	66,056	3,147	4,764.2
Colombia	48,258,494	1,299,613	2,693.0

a Data processed for the Department of Bolívar by the UNIMOL Laboratory [Indicated in bold]. Cutoff 30 November 2020.

Additionally, a higher frequency of infection was found in males (51.4%) than in females (48.6%); however, this difference was not statistically significant at *p > 0.05*, a behavior similar to that reported nationally, where 50.47% of cases were presented in males and 49.53% in females [16], and worldwide (males 51% and females 49%) [17], so that the male gender appears to present a higher risk of infection against SARS-CoV-2 by alteration in immunological factors, as reported in the literature [10, 16, 18].

Of the total cases, 60% occurred in subjects aged 20–49 years, as they are subjects who regularly move to their work and economic activities [8, 16], which is consistent with what happened in the most affected Departments of Colombia, where the frequency of cases for subjects aged 20–49 was 58.9% (Atlántico), 61.6% (Nariño), 63.2% (Cundinamarca), 65.65 (Antioquia), 70.2% (Meta), 61.2% (Valle del Cauca) and 55.6% (Amazonas). At the national level, this same population group presented a frequency of cases of 61.6% [16].

The highest proportion of cases with age ≥40 years occurred in males; in contrast, in the group with age ≤30 years, cases were more frequent in females (Figure 3), and the frequencies of cases for groups 0–10 and ≥80 years were the lowest as a result of the restrictions established on these groups since the beginning of the pandemic by the Departmental and National Health authorities [10, 11]. The reduced number of cases in children is also related to the low probability of infection that they present against the virus [19, 20].

Between May and June, the greatest number of cases was found in Bolívar (Figure 6), because the search for cases throughout the Department intensified. At that critical point of the pandemic the timely reporting of cases made by our surveillance network against COVID-19 was essential for the authorities to make the relevant regulations such as limiting the circulation of people and establishing epidemiological blockades in municipalities and neighborhoods with the highest number of cases, reducing the contagion capacity of the virus by limiting the circulation of infected people and social interactions with their family group. Thanks to this, Bolívar was one of the first Departments of Colombia to reach the stability of the epidemiological curve (Figure 8) and the logarithmic plateau (Figure 9) [21].

The municipalities of Bolívar with the greatest impact were the Cartagena capital for being the epicenter of the pandemic and the commercial and industrial center of the region, followed by Carmen de Bolívar, Magangué, Turbaco, Arjona and Santa Rosa de Lima, municipalities that have greater daily commercial and labor markets communication with Cartagena (Figure 5). However, Comparing the number of cases positive and cumulative incidence x100 inhabitants found in Cartagena with other capital cities of Colombia (Table 2), low impact is evidenced both in cases and cumulative incidence; the impact of COVID-19 in Cartagena was also below the main capital cities of the Caribbean Coast Colombian, such as Barranquilla, Valledupar and Monteria [16, 22]. Like the rest of the Department of Bolívar, Cartagena had a low negative impact on the number of cases reported thanks to the timely action of the strategic network against COVID-19.

**Table 2:** Cases and cumulative incidence in the main cities of Colombia [22].

Capital City	Population	Confirmed cases	Incidence of cases/100 thousand inhabitants
Bogota	7,181,000	374,077	5,209.3
Medellin	2,533,424	121,483	4,795.2
Cali	2,252,616	77,992	3,462.3
Barranquilla	1,274,250	45,023	3,533.3
Bucaramanga	607,428	22,512	3,706.1
Ibague	541,101	21,773	4,023.8
Villavicencio	551,212	20,785	3,770.8
Neiva	347,501	19,669	5,660.2
Valledupar	490,075	19,421	3,962.9
Cucuta	777,106	18,956	2,439.3
Bello	522,264	18,752	3,590.5
Manizales	434,403	17,288	3,979.7
Monteria	505,334	16,806	3,325.7
**Cartagena**^a^	**1,028,736**	**6,305**	**612.9**

a Data processed for Cartagena by the UNIMOL Laboratory [Indicated in bold]. Cutoff 30 November 2020.

Another aspect to highlight was the low frequency of infection in health personnel (13.1%) who have been in the first line of response to the pandemic; therefore, they are at high risk of frequent exposure to patients with high virus loads [16, 17]. In health personnel, the female gender was mostly affected, with 9.43% of cases, compared to the male gender (*p < 0.05*), a panorama that could be related to the fact that approximately 70% of health personnel belong to female gender both nationally and globally [17]. In general, the proportion of cases in health personnel has not been as high as during the outbreaks of SARS and MERS**,** pathogens that presented heterogeneity in transmissibility and the appearance of superpropagation events, particularly in hospitals [23, 24].

SARS-CoV-2 had a lethality frequency in the general population of 1.3%; of these deaths, 0.92% occurred in people aged ≥60 years who presented comorbidities, which is consistent with the literature where subjects of advanced age showed higher mortality [16, 21, 25]. Of the most important comorbidities or risk factors that predispose fatal outcomes in patients with COVID-19, there are alterations in renal function, high concessions of C-reactive protein, hypertension, diabetes, myocardial infarction, Heart failure, lung disease, Estimated Glomerular Filtration (eGFR) y cancer [25].

The male gender had a higher frequency of mortality (0.9%) against the SARS-CoV-2 compared to the female gender in all age groups (Figure 11), aspects that could be related to immunological alterations that predispose the male gender to fatal outcomes, as well as geographic variations in infection rates. However, as previously described, the available data come from a relatively small number of subjects and countries [10, 16, 17]. This shows the need to generate updated and reliable data on the behavior of COVID-19 in each country and the development of strategic networks that allow its containment, reducing its negative impact on health and the world economy. Comparing the COVID-19 mortality data found in Bolívar with those reported for the 10 most affected departments in Colombia [16], there is evidence of a low number of deaths for Bolívar (Table 3), and according to the cumulative incidences of mortality/100,000 inhabitants, Bolívar is also in the lowest position, with Amazona, Bogotá and Santander in the first places with the highest incidence of deaths per 100,000 inhabitants. The number of total deaths from COVID-19 recorded for Colombia is 36,584, and in Latin America, the countries with the highest number of deaths are Brazil, Argentina, Chile and Ecuador [10, 16]. In Bolivar the mortality rate was the lowest and the occupation of intensive care units (ICU) did not exceed the installed capacity of the institutions providing medical services. All this was possible due to the interventions of restriction in the mobility of people and epidemiological fences established from the timely identification of active cases and areas of high contagion. Another important aspect was the timely socialization of prevention strategies such as the use of mouth caps, hand washing and disinfection, social distancing, selective isolation on high-risk groups (subjects with comorbidities, > 60 years, child population) and their compliance by individuals.

**Table 3:** Deaths and cumulative incidences of COVID-19 deaths in Bolívar and other Departments of Colombia [16].

Department	Population	Dead	Incidence of mortality/100 thousand inhabitants
Bogota	7,181,000	8,505	118.4
Antioquia	6,855,517	4,038	58.9
Valle del Cauca	3,789,874	3,467	91.5
Santander	2,185,000	2,013	92.2
Atlantic	2,342,265	1,455	62.2
Cundinamarca	2,792,877	1,412	50.6
Nariño	1,335,521	849	63.6
Goal	919,129	629	68.4
Amazon	66,056	123	186.2
**Bolivar**^a^	**1,909,460**	**101**	**5.3**
Colombia	48,258,494	36,584	75.8

a Data processed for the department of Bolívar by the UNIMOL Laboratory [Indicated in bold]. Cutoff 30 November 2020.

The frequency of mortality for health personnel in Colombia was 0.28% (n = 101), and for Bolívar, it was 0.02% (n = 2), with a substantial reduction in mortality in Bolívar for health personnel.

It is important to include in this manuscript that the control measures taken by all countries worldwide have reduced the transmissibility and the number of global cases; however, it is plausible to state that despite all these measures and active case finding, the real number of infected people worldwide is unknown, and the transmission rate could increase with the general economic opening, as has happened in European and Asian countries such as Spain, France, Germany, Italy, the United Kingdom, Japan, and China, among others [26, 27, 28]. Cartagena is a tourist city and was the gateway for the virus to enter the Department of Bolivar. With the opening of the airports and international travel, people infected with new strains of SARS-CoV-2 virus could arrive, mutant strains that could have a higher transmissibility and lethality rate than those currently circulating in Cartagena, Bolivar and Colombia. This leaves an uncertain future outlook and alarms are raised by what has happened in Europe and Asia. Therefore, it is recommended to continue with prevention and control measures until an effective and safe vaccine against SARS-CoV-2 infection is available and commercialized.

It is important to mention that in tropical or subtropical countries where there are other important effects such as malaria in the future, they will have a greater burden on the cost of living and public health due to the coexistence with COVID-19; Malaria and COVID-19 have similar aspects both in their symptomatology and molecular diagnosis that it could be complex to differentiate by medical personnel, additionally, these two diseases have shown a potential to influence that would aggravate the health status of patients [29].

This study suffers from the usual limitations of initial investigations of infections by newly emerging pathogens.

## 5 Conclusion

The virus spread in 36 of the 46 municipalities of the Department of Bolívar, however, its negative impact in most municipalities was low (frequency of cases <0.31% and mortality <0.17%). Cartagena was the most affected in number of cases and deaths in Bolivar (frequency of cases 76.52% and mortality 0.97%), although at the national level it was the least affected of the main cities of Colombia and of Caribbean Colombian. The reduction in the impact of COVID-19 with respect to the number of cases and deaths in Cartagena and Bolívar, was due to the networking done between government, investigation and health entities of the Department of Bolívar. This has made it possible to keep the economic sector open while maintaining biosecurity protocols.

The highest number of cases occurred in subjects aged between 20 and 49 years regardless of sex. The male gender had a higher percentage of cases and deaths in all municipalities, and the highest mortality rate was found in subjects aged ≥60 years and with comorbidities. Infection in health personnel was low in general, however, the infection was mayor in females that in men, and the children had a low frequency of infection.

## Declarations

### Author contribution statement

Fabián Espitia-Almeida: Analyzed and interpreted the data; Wrote the paper.

Ramiro Pereira-Lentino: Contributed reagents, materials, analysis tools or data.

Juan Quintero-Soto: Performed the experiments.

Doris Gómez-Camargo: Conceived and designed the experiments; Contributed reagents, materials, analysis tools or data; Wrote the paper.

### Funding statement

This work was supported by National Institute of Health (Instituto Nacional de Salud-INS) of Colombia, University of Cartagena, Group UNIMOL; Departmental Laboratory of Public Health of Bolivar (Government of Bolivar); Administrative Department of District Health; Ports of Cartagena Society, Santo Domingo Foundation and REFICAR.

### Data availability statement

Data associated with this study has been deposited at Mendeley Data: https://doi.org/10.17632/bk3g62nfcv.1.

### Competing interest statement

The authors declare no conflict of interest.

### Additional information

No additional information is available for this paper.

## Appendix A Supplementary data

Supplementary Material.xlsx Supplementary Material.xlsx
